# Parity, Age at First Birth, and Risk of Death from Non-Hodgkin’s Lymphoma: A Population-Based Cohort Study in Taiwan

**DOI:** 10.3390/ijerph120809131

**Published:** 2015-08-05

**Authors:** Brian K. Chen, Chun-Yuh Yang

**Affiliations:** 1 Department of Health Services Policy and Management, Arnold School of Public Health, University of South Carolina, Columbia, SC 29201, USA; E-Mail: BCHEN@mailbox.sc.edu; 2 Department of Public Health, College of Health Sciences, Kaohsiung Medical University, Kaohsiung, No. 100 Shih-Chuan 1st RD, Kaohsiung 80708, Taiwan

**Keywords:** non-Hodgkin’s lymphoma, parity, mortality, cohort study

## Abstract

We undertook this study to examine whether there exists an association between parity and age at first birth and risk of death from non-Hodgkin’s lymphoma (NHL). Our sample included a total of 1,292,462 women who had a first and singleton childbirth between 1 January 1978 and 31 December 1987. We followed each subject from their first childbirth to 31 December 2009, and determined their vital status by merging natality data with Taiwan’s national death certificate database. Hazard ratios (HR) of death from NHL associated with parity and age at first birth were estimated using Cox proportional hazard regression models. In all, 412 NHL deaths were recorded during 34,980,246 person-years of follow-up. NHL mortality rate was 1.18 cases per 100,000 person-years. Older age at first birth (>23 *vs.* ≤23 years) was linked to an increased risk of death from NHL (adjusted HR = 1.41; 95% CI = 1.13–1.75). Controlling for age at first birth, the adjusted HR were 0.74 (95% CI = 0.55–0.98) for women with 2 births, and 0.71 (95% CI = 0.53–0.95) for women with 3 births or more, respectively, when compared with women with only 1 birth. A statistically significant downward trend in the adjusted HR for NHL death was detected with increasing parity (p for trend = 0.05). The HR of death from NHL was decreased by 7% (HR = 0.93; 95% CI = 0.87–0.99) for each additional parity. Our findings are consistent with reproductive factors (parity and early age at first birth) conferring a protective effect against the risk of NHL death.

## 1. Introduction

In Taiwan, non-Hodgkin’s lymphoma (NHL) is the 9th leading cause of cancer mortality for males and females [1]. The age-adjusted mortality rate for NHL was 3.7 per 100,000 among males and 2.5 among females in 2013 [1]. The age standardized incidence rate of NHL is 7.76 per 100,000 among males and 6.04 among females in 2011 [2]. The incidence of NHL varies from around 2 to 3 per 100,000 in Thailand and China to about 14 per 100,000 among whites in America and Canada [3].

Worldwide, the incidence of non-Hodgkin’s lymphoma (NHL) has experienced an upward trend [4,5]. The etiology of NHL, while largely unknown, may be associated with sex hormones. It has been shown that the incidence rates for NHL are commonly higher in men than in women [3]. Aside from their role in endocrine function, sex hormones also play a part in immune system regulation and could therefore be involved in lymphomagenesis [6,7,8].

It is biologically plausible to posit a relationship between reproductive factors and NHL mortality risk, since serum estrogen levels may rise approximately 100-fold during pregnancy [9], and high levels of estrogen exposure are associated with reduced interleukin-6 secretion, a growth factor for intermediate- and high-grade NHL [10,11,12,13].

Nonetheless, the role of reproductive factors (parity and age at first birth) in the etiology of NHL in women has garnered only limited attention in the medical literature, and the results have been mixed. Some studies found an inverse relationship between increasing parity and NHL risk [14,15,16] and others reported no association [17,18,19,20,21,22,23,24]. Similarly, reported associations between older age at first birth and NHL risk have been inconsistent, with two studies finding an increased risk [16,25] and another study reporting a decreased risk [22]. Furthermore, other studies failed to establish any association [14,15,17,18,19,20,21,23,24].

Although the general results are inconsistent, previous studies do indicate a possible role for selected reproductive factors, particularly parity, in the reduction of NHL risk in women. We conducted a study on a large cohort of women who experienced a first and singleton childbirth between 1 January 1978 and 31 December 1987 and subsequent NHL mortality risk in order to better understand the association between parity, age at first birth, and the risk of death from NHL in Taiwan.

## 2. Materials and Methods

### 2.1. Data Source

Taiwan has excellent records of vital statistics, including births and deaths. By law all parents in Taiwan are required to register at a local household registration office within 15 days. The Birth Registration System, under management by the Department of Interior, has released electronic data on live births since 1978. The official registration form, which includes information on maternal age, education, parity, gestational age, date of delivery, infant gender, and birth weight, must be completed by the physician attending the delivery. Because registration of births is mandatory, and because most birth certificates are completed by obstetricians since the great majority of deliveries in Taiwan occur at a licensed medical facility [26], Taiwan’s birth registration data are considered complete, reliable and accurate. Furthermore, all live births must be registered at local household registration offices in order to acquire the child’s citizenship (every citizen gets a unique identification number at birth). We have used this dataset in previous studies [27,28]. In addition, this study was approved by the ethics review board of the Kaohsiung Medical University Hospital (KMUH-IRB-20130042).

### 2.2. Study Population

We identified all women with a first and singleton childbirth in the Birth Registry between 1 January 1978 and 31 December 1987. During this period, there were 1,333,312 first singleton births in Taiwan. Data on all subsequent births were also culled from the Birth Registry. Of the 1,399,312 so-identified primiparous women, 106,850 subjects were excluded because data were missing for at least one or more study variables, such as maternal age (n = 100,099), years of schooling (n = 382), marital status (n = 2665), or place of birth (n = 4535). Exclusion of women with missing data elements resulted in a sample of 1,292,462 women for the analysis. Details on this sample have previously been described in an earlier publication [29].

### 2.3. Follow-up

Each study subject is identified by a unique identification number. Using this number, we followed each subject from the time of her first childbirth to 31 December 2009, and the vital status of all study subjects was ascertained by merging records with Taiwan’s mortality database, which includes the date of any deaths occurring in this cohort as of the end of the study period. Of the 1,292,462 women in the sample, none had a missing personal identification number; therefore, no study subject was lost to follow-up. As with birth certificates, it is also mandatory to register all deaths at local household registration offices, so statistics on mortality in Taiwan are considered to be highly accurate and complete [29,30].

### 2.4. Statistics

The number of person-years of follow-up for each subject was determined from the date of first childbirth to the date of death or 31 December 2009. Mortality rates were calculated by dividing the number of NHL deaths by the number of person-years of follow-up. We used Cox proportional hazard regression models with time-dependent covariates to estimate the hazard ratios (HRs) of NHL mortality associated with parity (the total number of births recorded in the last childbirth record for each subject in the follow-up period) and maternal age at first birth, and recorded the 95% confidence intervals (CIs) for the HRs. NHL death was identified by codes 200 or 202 of the International Classification of Disease, Injury, and Causes of Death (9th revision). The variables in the final model comprised age at first birth (≤23, >23), parity (1, 2, 3 or more), marital status (married, unmarried), years of schooling (≤9, >9 years), and place of birth (hospital/clinic, home/other). We assessed and found no violations for the proportional hazards assumption with respect to all variables. We used two-sided tests for all statistical analyses, and considered p values of less than 0.05 to be statistically significant. All analyses were performed using SAS (version 9.2, SAS Institute Inc., Cary, NC, USA).

## 3. Results and Discussion

Altogether, 1,292,462 primiparous women with the complete set of dependent and independent variables were included in the analysis. A total of 34,980,246 person-years were observed during the follow-up period, beginning at the time of each subject’s first childbirth to the earlier of her death or 31 December 2009. There were 412 NHL deaths, corresponding to a mortality rate of 1.18 per 100,000 person-years.

In Table 1, we present person-years and NHL deaths by age at recruitment (maternal age at first childbirth), parity, marital status, years of schooling, and birth place. The mortality rates were, respectively, 1.63 per 100,000 person-years among women with a single childbirth, 1.15 per 100,000 person-years among those with 2 childbirths, and 1.08 per 100,000 person-years among with 3 or more childbirths.

**Table 1:** Demographic characteristics of the study cohort.

Variables	No. of Subjects	Follow-up Person-Years	No. of Death from Non-Hodgkin’s Lymphoma	Mortality Rate (per 100,000 Person-Years)
Age at Recruitment (1st birth)				
≤23	551,759	15,312,470.08	149	0.97
>23	740,703	19,667,775.92	263	1.34
Parity				
1	157,207	4,170,772.33	68	1.63
2	564,727	15,124,112.33	174	1.15
3+	570,528	15,685,361.33	170	1.08
Marital Status				
Married	1,260,615	34,115,479.25	399	1.17
Not married	31,847	864,766.75	13	1.50
Years of Schooling				
≤9 years	722,518	19,850,938.17	225	1.13
>9 years	569,944	15,129,307.83	187	1.24
Birth Place				
Hospital/clinic	1,245,925	33,638,862.83	390	1.16
Home/other	46,537	1,341,383.17	22	1.64

The HRs adjusted for multiple covariates and 95% CIs are shown in Table 2. An older age at first birth (>23 *vs.* <23 years) was associated with an increased NHL mortality risk (adjusted HR = 1.41; 95% CI = 1.13–1.75). After controlling for age at first birth, the adjusted HR were 0.74 (95% CI = 0.55–0.98) for women who had 2 children, and 0.71 (95% CI = 0.53–0.95) for women with 3 or more births, respectively, compared to women who had only 1 child. The downward trend in the adjusted HR for NHL mortality was statistical significant with increasing parity (*p* for trend = 0.05)

As far as we are aware, our study represents the largest cohort (n = 1,292,462) to date that examines the relationship between reproductive factors (parity and age at first birth) and NHL mortality risk. Our prospective cohort study found an inverse association between parity and the risk of death from NHL. This findings of a reduced risk of death from NHL with higher parity is consistent with part of the existing literature [14,15,16], but is in contradiction with other studies that reported no association of NHL risk with parity [17,18,19,20,21,22,23,24].

**Table 2:** Association between parity, age at first birth, and relative risk of death from non-Hodgkin’s lymphoma over a 32-year follow-up period.

Variables	Crude RR (95% CI)	Multivariate-Adjusted RR * (95% CI)
Age at recruitment (1st birth)		
≤23	1.00	1.00
>23	1.45 (1.18–1.77)	1.41 (1.13–1.75)
Parity		
1	1.00	1.00
2	0.70 (0.53–0.93)	0.74 (0.55–0.98)
3+	0.64 (0.49–0.85)	0.71 (0.53–0.95)
	*p = 0.01 for linear trend*	*p = 0.05 for linear trend*
Continuous	0.89 (0.83–0.96)	0.93 (0.87–0.99)
Marital status		
Married	1.00	1.00
Not married	1.27 (0.73–2.21)	1.21 (0.70–2.12)
Years of schooling		
≤9 years	1.00	1.00
>9 years	1.14 (0.94–1.38)	1.03 (0.84–1.27)
Birth place		
Hospital/clinic	1.00	1.00
Home/other	1.31 (0.85–2.02)	1.46 (0.94–2.26)

* Mutually adjusted.

The biological mechanisms by which increased parity may confer protection against NHL in women remains unanswered. Pregnancy raises serum estrogen levels nearly 100-fold [9]. As a result, increasing parity likely results in an increase in total lifetime exposure to estrogen. Both experimental and observational studies demonstrated that estrogen plays a direct role in the etiology of NHL, as estrogen inhibits interleukin-6 secretion, which has been posited as a potent growth factor for intermediate- and high-grade NHL [10,11,12,13]. Therefore, if estrogen is associated with a reduction in NHL risk, it is possible that multiple pregnancies and the resulting increase in serum estrogen levels could offer some protection against NHL. Our analysis provides support for this hypothesis.

In this prospective cohort study, we found a 1.41-fold increase in NHL mortality risk for women who had their first birth at age 23 years or later, relative to those who had their first birth before age 23 years after controlling for parity. This result agrees with two prior studies [16,25] but not with other studies that either reported a reduction in risk [22] or no association with older age at first birth [14,15,17,18,19,20,21,23,24]. The reason for the increase in risk with first pregnancies at an older age is unknown. A possible explanation is that a younger age at first birth plays a protective role against developing NHL through elevated levels of some hormones (including estrogen) that are not as effective at older ages. It has been found that the age of menarche (and thus exposure to menstrual estrogen stimulation) is positively associated with the age at which a women delivers her first child [31]. Earlier exposure to regular menstrual cycles and the resulting increase in exposure to estrogen may protect against tumor initiation, supporting a positive association between earlier age at first birth and NHL risk reduction [10,11,12,13]. Our finding of an increased risk of death from NHL with older age at first birth is consistent with the hypothesis that estrogen exposure is protective with respect to NHL risk. Nonetheless, because no consistent evidence to date exists to validate an association between age at first birth and NHL risk, the possibility that this is a chance finding must also be considered. Future work is required before we can find a definitive answer concerning the impact of age at first birth on the risk of death from NHL.

Despite their inherent limitations, mortality data have been widely used in the academic literature to study epidemiologic hypotheses. Of particular importance in the evaluation of the completeness and accuracy of the mortality data before any conclusion based on an analysis of such data is made. Taiwan’s death certification registry offers a particularly attractive opportunity to study epidemiological questions using mortality data. In Taiwan, the decedent's family is required to obtain a death certificate from the hospital or local community clinic in order to have the decedent’s body buried or cremated. Death certificates must be completed by physicians in Taiwan. It is also mandatory to register all deaths at local household registration offices, so the death registration is accurate, reliable and complete. Furthermore, the uncensored nature of Taiwan’s mortality database offers the researcher an opportunity to follow study subjects using unique identification numbers and eliminate selection bias. The recall bias characteristic of survey data is also unlikely to be a significant factor for parity and age at first birth.

In 1995, Taiwan inaugurated its National Health Insurance (NHI) program to extend health insurance coverage to all residents of Taiwan. As of 2007, the NHI program attained near universal coverage with a coverage rate of 98.4%, providing insurance to 22.60 million out of 22.96 million Taiwanese [32]. As a small island with a convenient communication network, we believe that nearly all NHL patients had access to healthcare in the post-NHI period. In Taiwan, the overall 5-year survival rate for NHL is approaching 50% [5]. However, there is no reason to believe that there would be any selection bias created by the 5-year survival rate to confound the relationship between NHL mortality and reproductive factors (parity and age at first birth). We therefore believe that the use of mortality data rather than inpatient admissions data should not bias the associations that we observed.

A few potential limitations of this study should be noted. First, the death-certificate database used in this study does not specify the subtype of NHL cause of death. As a result, we were not able to estimate the risk of death from NHL in greater detail, relative to the risk for specific NHL subtypes, which is a necessary analysis given the heterogeneity of the disease. Furthermore, by studying NHL mortality rather than incidence, our study may have been more likely to capture specific subtypes (e.g., more aggressive rather than the indolent subtypes). Second, Taiwan’s birth registry includes only live births and excludes stillbirths and abortions, precluding an examination of the possible role of gravidity in NHL mortality risk. Third, our study focused solely on mortality among parous women. We did not examine the possible role of nulliparity on the risk of death from NHL, limiting the generalizability of our findings. Fourth, the number of children born to each woman was available only for those who had a first live birth between 1 January 1978, and 31 December 1987. The mean age at first birth was 24.33 years in our cohort. This value was increased during the study period (ranged from 23.3 to 28.1 in 2006) [33]. Contrarily, the total fertility rate was decreased during the study period (ranged from 2715 ‰ in 1978 to 1115 ‰ in 2006) [33]. The generalizability of our findings is thus also limited. Fifth, we note that several studies have found an association between hormone replacement therapy (HRT) and a decreased risk of NHL [20,21,23,34,35,36,37]. Other studies, however, have failed to find such an association [24,38,39]. In addition, previous studies have largely reported no association between the use of oral contraceptives (OCs) and NHL risk [15,24,35]. In this study, we were unable to adjust for these two hormonal factors due to data limitations. Nevertheless, since the use of OCs and HRT are low in Taiwan compared with Western countries [40,41], the confounding effect resulting from not controlling for these two factors should be, at most, extremely limited. Given the low use of OCs and HRT in Taiwan, we were able to assess parity and age at first birth with limited influence from exogenous hormones. Finally, omitted variable bias is another limitation; we lacked data on other hypothesized occupational and environmental risk factors, such as exposure to benzene, gasoline, and phenoxy acids and chlorophenol herbicides [5,42,43]. Nevertheless, the potential for confounding socioeconomic factors should be evaluated. Age at first birth can be explained at least in part by socioeconomic differences, with age at first birth considerably younger among those with lower levels of education. We addressed this potential confounder by including years of schooling as a proxy for socioeconomic status in the multivariate analysis.

## 4. Conclusions

In conclusion, our results showed that increasing parity is associated with decreasing risk for death from NHL mortality. We further found an association between older age at first birth and risk of NHL. These results are consistent with the hypothesis that reproductive factors (parity and age at first birth) that increase hormonal exposure, which limits the secretion of a potent growth factor for lymphoma, may confer a protective effect against the risk of death from NHL.
